# Advances in genome editing in plants within an evolving regulatory landscape, with a focus on its application in wheat breeding

**DOI:** 10.1007/s13562-025-00981-w

**Published:** 2025-04-15

**Authors:** Natasha Brock, Navneet Kaur, Nigel G. Halford

**Affiliations:** Rothamsted Research, Harpenden, AL5 2JQ UK

**Keywords:** Biotechnology, CRISPR/Cas, Crop improvement, Oligonucleotide-directed mutagenesis, Risk assessment and regulation, Site-directed nucleases, TALENs

## Abstract

Population growth, diminishing resources and climate change are some of the many challenges that agriculture must address to satisfy the needs of the global population whilst ensuring the safety and nutritional value of our food. Wheat (*Triticum aestivum*) is tremendously important for human nutrition, providing starch (and, therefore, energy), fibre, protein, vitamins, and micronutrients. It is the second most widely grown crop behind maize (*Zea mays*), with 808 million tonnes of grain being produced in 2021–2022. In comparison, the production figure for 1961 was 222 million tonnes, and there have been similar increases for maize and rice (*Oryza sativa*). World population over the same period has increased from just over 3 billion to just over 8 billion, a stark reminder of just how important increased crop production has been in maintaining food security over that period, and for these cereals it has been achieved without additional land use. Plant breeding has played an important part in enabling crop production to keep increasing to meet demand and this will have to continue through the coming decades. Innovative technologies will play a part in that, and here we review how the new technology of genome editing is being applied in crop genetic improvement, with a focus on wheat. We cover oligonucleotide-directed mutagenesis and the use of site-directed nucleases, including meganucleases (MegNs), zinc-finger nucleases (ZFNs), transcription activator-like effector nucleases (TALENs), and clustered regularly interspaced short palindromic repeats (CRISPR)-associated (Cas) nucleases. We describe established genome editing strategies, mainly involving gene ‘knockouts’, and the new applications of base and prime editing using CRISPR/Cas. We also discuss how genome editing for crop improvement is developing in the context of an evolving regulatory landscape.

## Introduction

Crop improvement has been an essential focus for humanity since land was first farmed and cultivated 10,000 years ago in the fertile crescent. For most of this period the methodology was very basic, and while it involved artificial selection and genetic inheritance, the mechanisms underpinning those processes were not elucidated until the nineteenth and twentieth centuries. Plant breeding was finally put on a scientific footing thanks to the work of Mendel from 1857 onwards. Mendel’s work was published but largely overlooked until its rediscovery at the beginning of the twentieth century. The application of science-based plant breeding, along with the mechanisation of agriculture and the development of chemical fertilisers, herbicides and pesticides enabled huge improvements to be achieved in crop yield and production. This accelerated from the early 1960s onwards, largely due to the adoption of high yielding, dwarf varieties of cereals. These varieties were less prone to lodging and partitioned more of their assimilated carbon and nutrients into grains instead of stems and leaves. The effect was so dramatic that it was labelled the ‘Green Revolution’ (Hamdan et al. 2022).

**Fig. 1:**
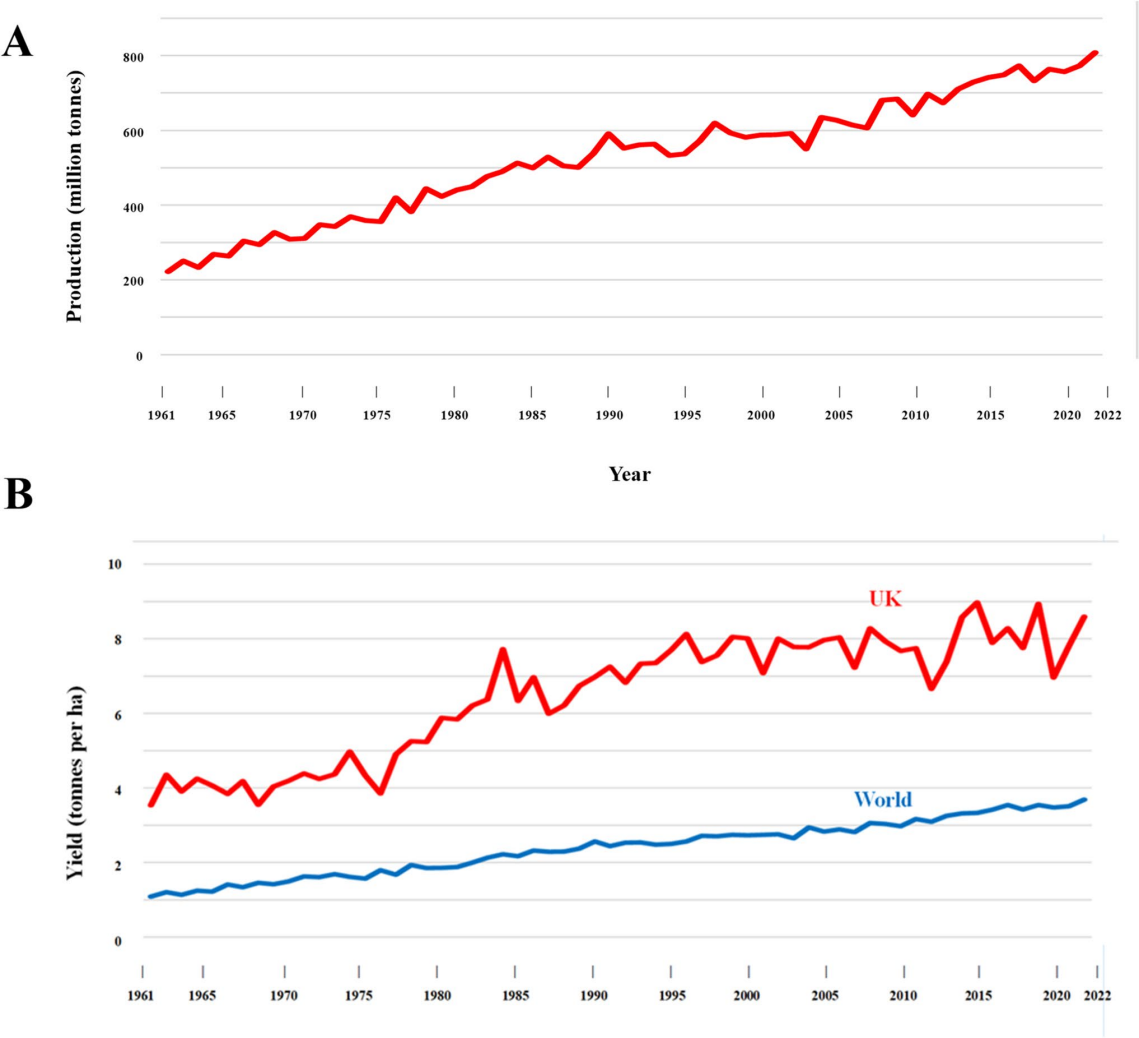
**A** Global wheat production from 1961 to 2022. **B** Wheat yield for the world and the UK, 2011 to 2022. Data from United Nations Food and Agriculture Organisation

**Fig. 2:**
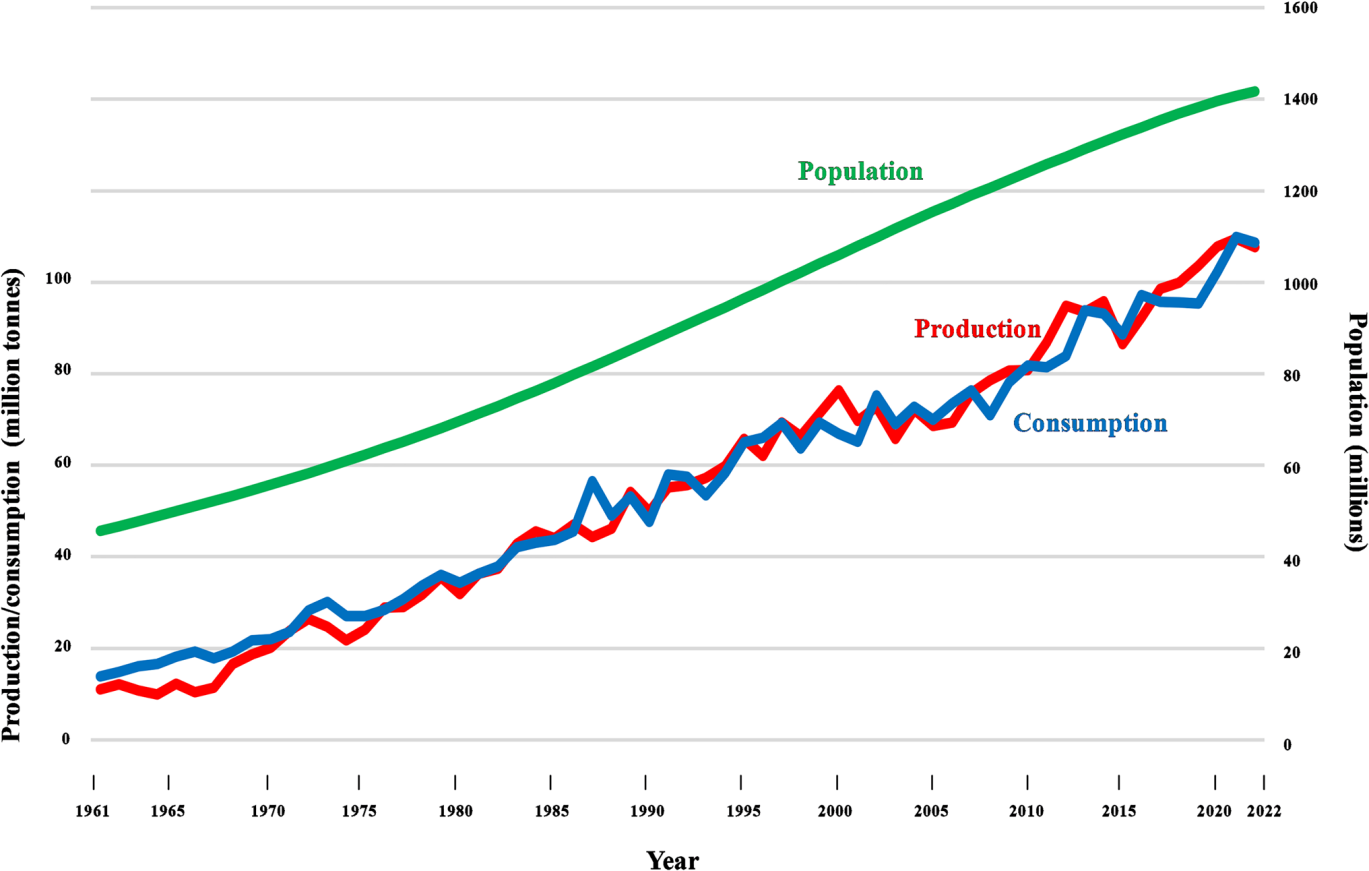
Indian population, growth in population, wheat production and wheat consumption from 1961 to 2022. Population data from the United Nations; wheat data from the United Nations Food and Agriculture Organisation

Figure 1A shows global wheat (mainly *Triticum aestivum*) production in the period from 1961 to 2022, during which it increased from 222 million tonnes to 808 million tonnes. Maize (*Zea mays*) and rice (mainly *Oryza sativa*) production has seen similar increases, from 205 to 1160 and 216 to 776 million tonnes, respectively. These increases in production were brought about by increases in yield rather than growing area, with world wheat yield improving from just over 1 tonne per hectare in 1961 to just under 4 tonnes per hectare in 2022 (Fig. 1B). Even developed countries, such as the UK, where yields were already relatively high, managed to double yield over this period, although yields this century do appear to have plateaued (Fig. 1B). This was a great and possibly underappreciated achievement: world population over the same period increased from just over 3 billion to just over 8 billion and it is sobering to consider what would have happened if those yield increases had not been achieved. This is illustrated in Fig. 2, which shows population growth, wheat production and consumption in India as an example. The population of that country more than trebled over that period, from 456 million to 1.417 billion. Wheat consumption increased even more, from just over 10 million tonnes to over 100 million tonnes, driven not only by the population increase but also by *per capita* consumption, which rose from approximately 20 kg in 1961 to 70 kg in 2022. However, the huge increase in demand that resulted was matched by an approximately tenfold increase in production.

**Fig. 3:**
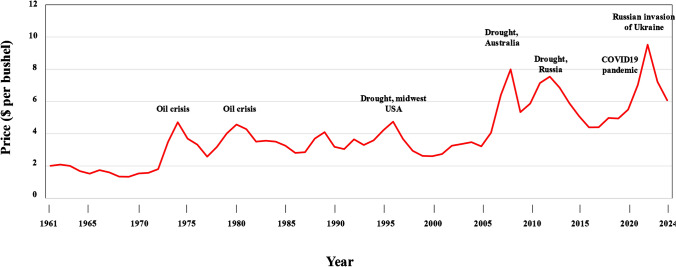
Global wheat trading price from 1961 to 2022. Data: United States Department of Agriculture. The timings of extreme weather and other events affecting major wheat exporting countries are indicated

The relationship between wheat supply and demand does fluctuate, of course, and this is reflected in the world market price. Figure 3 shows United States Department for Agriculture (USDA) data on the global wheat trading price from 1961 to 2024 (https://apps.fas.usda.gov/psdonline/app/index.html#/app/home). The price in 1961 was approximately $2 per bushel ($73.50 per tonne), while it is currently (2024) around $6 per bushel ($220 per tonne). However, the graph shows a number of peaks caused by weather and political issues, the highest being in 2022 when food security concerns caused by the global COVID19 pandemic of 2020 to 2022 and Russia’s invasion of Ukraine pushed the price close to $10 per bushel ($367 per tonne). Extreme weather events like the droughts in the USA, Australia and Russia that are marked in Fig. 3 are becoming more frequent and more severe with climate change. Indeed, the UK suffered one of its wettest springs on record in 2024. Price spikes have the severest consequences for the most vulnerable people, increasing levels of poverty, hunger and malnutrition.

Climate change represents a huge challenge for agriculture in the coming decades, due to the widespread nature, potential severity and unpredictability of its impact. Will drought be the major problem in a particular area or flooding, or heat stress? Plant breeders need to think many years ahead, but there are large uncertainties in the climate projections of Global Climate Models (IPCC 2021). Agriculture also faces the challenges of competition for land use, insufficient fresh water supply, labour shortages and costs, loss of crop protection chemistry due to political interference, coupled with lack of investment in new crop protection chemistry, soil degradation, increasing costs of mineral fertilisers, and a demand from governments to decrease its carbon footprint, much of which is accounted for by nitrogen fertiliser production. The yields of wheat in the UK in recent decades illustrated in Fig. 1B have been heavily dependent on the availability of cheap nitrogen fertiliser and the ability to prevent or control disease outbreaks and insect damage, but the era when farmers were able to apply nitrogen fertiliser, herbicides, fungicides and pesticides whenever they were required may already be over. At the same time, agriculture is expected to supply a growing population that is more aware than ever of food safety, nutritional value and quality issues, as well as the increasing demand for its products for non-food uses, including bioethanol, biodiesel and industrial starch production.

The aim of this preamble is to convince the reader that there can be no complacency when it comes to the continuing need for crop improvement, and that plant breeders will need to use all the tools available to them if they are to develop new varieties quickly enough. Sadly, this does not seem to resonate with political leaders in many parts of the world, and in some cases new genetic technologies have faced regulatory barriers so obstructive as to prevent their development and application at all. The risk assessment and approval system imposed by the European Union (EU) on genetically modified (GM) crops, for example, is so tortuous that no-one has even applied for permission to commercialise a GM crop for cultivation in the EU for a decade and a half. That is not to say that the EU does not consume GM crops: it imports millions of tonnes of GM soybean, maize, and cotton, the first two mainly for use in animal feed. It is much easier to get approval to import GM crop products into the EU for food and feed use because the EU animal feed industry needs imported soybean and maize. These products enter the food chain, of course, so presumably the EU regulators agree that there is no safety issue with them. Yet its regulations and processes have utterly stymied the development of GM crops for cultivation in Europe for a generation.

GM is a twentieth century technology and can no longer be described as new. Genome editing, on the other hand, is little more than a decade old and is still being developed at a rapid rate. The main focus of this review is the development of this new technology and its application to crop improvement, with a focus on wheat, in the context of a complex and shifting regulatory landscape.

## Genome editing, a second revolution in biotechnological crop improvement

If GM was the first biotechnological revolution in crop improvement, then genome editing could be described as the second. Genome editing is an umbrella term covering a range of techniques that enable specific changes to be made to target genes; in other words, it can be viewed as targeted mutagenesis. Some involve a genetic modification step, but even when that is the case, the transgenes that are used can be segregated away once the editing has been done, resulting in a plant in which no transgenes are present but a native gene has been ‘edited’. These technologies can be divided into those that involve oligonucleotide-directed mutagenesis (ODM) and those that involve site-directed nucleases, such as meganucleases (MegNs), zinc-finger nucleases (ZFNs), transcription activator-like effector nucleases (TALENs), or CRISPR-associated (Cas) nucleases, which originated from a bacterial defence system based on clustered regularly interspaced short palindromic repeats (CRISPR).

### Oligonucleotide-directed mutagenesis (ODM)

The oligonucleotides used in ODM are usually single-stranded DNA molecules between 20 and 100 nucleotides in length, but there are variants, including the use of double-stranded RNA–DNA oligonucleotides (RDOs), and small fragment homologous replacement (SFHR), which uses longer (> 200 nucleotides) single- or double-stranded DNA molecules. The oligonucleotide, whichever type is used, is designed to be identical in nucleotide sequence to a target gene in the plant apart from a small number (usually between 1 and 5) of ‘mismatches’ where the nucleotide sequence is different. These are the mutations that are to be introduced into the genome.

The oligonucleotide can be delivered into protoplasts after treatment with polyethylene glycol or electroporation, or into cultured plant cells by particle bombardment. Once delivered, it binds to the complementary sequence of nucleotides in the target gene. The plant’s own DNA repair machinery then replaces nucleotides where the mismatches occur, sometimes in favour of the mutations carried by the oligonucleotide, or replaces the native stretch of DNA with the oligonucleotide.

Importantly, the United States Department for Agriculture, Health Canada and other regulatory authorities have classified ODM as a mutagenesis rather than GM technique. The first example of its application in a commercial crop that we are aware of was its use by Cibus to produce a herbicide-tolerant oilseed rape (Canola) (*Brassica napus*) variety. The variety is used in combination with BASF’s CLEARFIELD® Production System, which is based on imidazolinone herbicides. These herbicides inhibit the action of acetolactate synthase (ALS) and, therefore, the synthesis of branched side-chain amino acids (valine, isoleucine and leucine). The trait was produced by introducing single nucleotide mutations in two *ALS* genes, with the enzymes encoded by those genes remaining active but no longer bound by the inhibiting herbicide. In 2014, a full decade ago, Health Canada notified Cibus that it had no objection to the food use of the oilseed rape variety carrying the trait, effectively clearing the way for commercial cultivation.

### Site-directed nucleases (meganucleases, zinc finger nucleases, TALENS and CRISPR/Cas9)

Genome editing with site-directed nucleases (SDNs) (also known as sequence-specific nucleases) also exploits the cell’s own DNA repair mechanisms. Normally these mechanisms repair double-stranded breaks in DNA caused by, for example, ionising radiation or chemical mutagens. Cells have multiple mechanisms for repairing double-stranded breaks in DNA, some of which are error-prone, and it is the tendency of the cell’s DNA repair machinery to introduce mutations into the DNA sequence that is exploited in genome editing using SDNs.

Four classes of SDNs are now available for use in genome editing. Three of these, meganucleases (MegNs), zinc finger nucleases (ZFNs) and transcription activator-like effector nucleases (TALENs) target the correct sequence via protein-DNA interactions. Once the sequence has been targeted, the DNA is cleaved by the nuclease domain and subsequently repaired by the plant’s own DNA repair mechanisms.

### Meganucleases (MegNs)

MegNs are naturally-occurring restriction enzymes derived from microbes. The DNA recognition sequence is typically from 12 to 40 bp long, and MegNs can be engineered to recognise different target sites, so they can be directed to specific target genes. However, the DNA recognition domain of the protein overlaps with the nuclease domain, so engineering changes in target site specificity may affect the ability of the enzyme to cut DNA. This has been overcome by some researchers using sophisticated protein modelling techniques; however, the use of MegNs has largely been superseded by methods that are simpler to use. MegNs have been used successfully to edit crop species (Daboussi et al. 2015) but we are not aware of any crop varieties produced in this way making it to the market.

### Zinc finger nucleases (ZFNs)

ZFNs are artificially engineered restriction enzymes, comprising a fusion of the DNA-binding domain from the zinc finger class of transcription factors with the catalytic domain of the *Fok*I nuclease. An individual ‘finger’ in the zinc finger domain comprises thirty amino acids folded around a zinc ion. Each zinc finger binds a specific target site of three base pairs, which means that the overall binding specificity can be engineered by combining different zinc fingers into arrays. Further changes can be made by altering the individual amino acids in a finger, so that domains can be designed to target any DNA sequence. *Fok*I functions as a dimer, so two DNA recognition domains are separately fused with the *Fok*I nuclease domain in order to bring the two *Fok*I proteins together at the target site. ZFNs are considerably easier to design than MegNs but have a greater propensity for introducing off-target effects because their specificity depends not only on the target DNA sequence itself, but also on the surrounding region. This can lead to multiple breaks in the DNA, resulting in genome fragmentation and instability. Nevertheless, ZFNs have been used to edit the acetohydroxyacid synthase (*AHAS*) gene of wheat to confer resistance to the herbicide, imazamox (Ran et al. 2018). In maize, ZFNs were used to enable the site-specific integration of transgenes into a genomic locus to create multigene stacks (Kumar et al. 2015), while in tobacco (*Nicotiana tabacum*), a *GUS:NPTII* reporter gene has been edited to generate chromosome breaks (Wright et al. 2005). ZFNs were also used to edit the *ALS, SuRA* and *SuRB* genes to confer resistance to imidazolinone and sulphonylurea herbicides (Townsend et al. 2009). In rice, the *OsQQR* gene has been edited to enable the detection and identification of appropriate integration sites for genes at specific chromosomal locations for herbicide tolerance (Cantos et al. 2014). However, as with MegNs, we are not aware of any commercial crop varieties being generated using ZNFs.

### Transcription activator-like effector nucleases (TALENs)

In comparison to MegNs and ZFNs, TALENs are cheaper, more efficient and more specific, with fewer off-target effects and consequently reduced toxicity. Like ZFNs, TALENs are artificial fusions of a DNA binding domain and the catalytic domain of the *Fok*1 nuclease. In this case, the DNA binding domain comes from transcription activator-like effectors (TALEs), a family of proteins produced by plant pathogens in the genus *Xanthomonas*. During infection, TALEs enter a plant cell and activate expression of specific genes, typically resulting in the plant becoming more susceptible to colonisation by the pathogen. The DNA binding region of TALEs comprises approximately thirty tandem repeats of 33 to 35 amino acids, with sequence specificity imparted by just two variable amino acids called the repeat variable di-residue. Each repeat variable di-residue recognises a specific DNA sequence, so any gene can be targeted simply by assembling an array of repeats with the appropriate repeat variable di-residues. The downside of this is that TALENs are relatively large proteins.

The use of TALENs for genome editing of plants was demonstrated in 2013 (Zhen et al. 2013) and TALENs was used to produce the first crop variety that had been edited with a SDN to be released commercially. This variety, produced by Calyxst in the USA and called Calyno, is a high oleic acid soybean, in which genes involved in oleic acid desaturation, *FAD2-1A, FAD2-1B* and *FAD3A*, have been knocked out (Haun et al. 2014; Demorest et al. 2016). This prevents oleic acid, a monounsaturated fatty acid, from being converted to the polyunsaturated fatty acids, linoleic acid and α-linolenic acid, resulting in oleic acid making up about 80% of the oil from the edited soybean. Oleic acid is less prone to oxidation during storage than polyunsaturated fats, making it less likely to form compounds that affect flavour and give an ‘off’ colour and aroma. The usual method of preventing polyunsaturated fat oxidation involves chemical hydrogenation and this runs the risk of creating *trans* fatty acids. *Trans* fatty acids contain double bonds in a different orientation to the *cis* fatty acids present in natural plant oils and behave like saturated fat in raising blood cholesterol. Oil from high oleic acid soybeans does not require hydrogenation. High oleic acid soybeans are not new, but the ones that were already on the market had been made using RNA interference (RNAi), a GM technology. While genome editing can be used to knock a gene out and create a *null*, RNAi will only reduce the expression of a gene. Which technology is preferable will depend on the target trait, but producing crops where a trait has already been engineered successfully using RNAi and in which a clean knockout would be better is an obvious target for the first uses of genome editing in crop improvement.

TALENs has been used in wheat to knock out the *TaMLO* gene, conferring resistance to powdery mildew (Wang et al. 2014). In potatoes (*Solanum tuberosum*), *ALS* and an endogenous constitutive promoter have been edited to impart herbicide tolerance (Butler et al 2016; Forsyth et al. 2016) and a vacuolar invertase (*vlnv*) gene has been edited to produce low reducing sugar tubers with better storage stability and less potential for acrylamide formation during frying and roasting (Clasen et al. 2016). In sugar cane (*Saccharum officinarum*), TALENs has been used to target caffeic acid *O*-methyltransferase to reduce lignin and improve biofuel production (Jung and Altpeter 2016), while in rice, the *OsBADH2* gene has been edited to produce more fragrant rice (Shan et al. 2015). However, we are not aware of varieties carrying these traits being commercialised yet.

### Clustered, regularly interspaced, short palindromic repeat (CRISPR)/CRISPR-associated protein (Cas) (CRISPR/Cas)

The technique that has really brought genome editing to the fore uses the clustered, regularly interspaced, short palindromic repeat (CRISPR)/CRISPR-associated protein (Cas) nuclease system. While MegNs, ZFNs and TALENS rely on protein-DNA interactions to recognise target sites in DNA, the CRISPR/Cas system uses a guide RNA (gRNA) for targeting of the Cas nuclease. This is advantageous because it is much easier to design genes encoding gRNAs than genes encoding targeting domains in proteins. It is also easier to predict off-target effects and it is possible to introduce many edits at once by using multiple gRNAs in the same experiment, known as multiplexing.

CRISPR/Cas is a bacterial immune system discovered by Jennifer Doudna at the University of California, Berkely, and Emmanuelle Charpentier at Umeå University in Sweden, and colleagues (Jinek et al. 2012), resulting in Doudna and Charpentier being awarded the Nobel Prize for Chemistry in 2020. The system confers resistance to foreign genetic elements such as those of invading phages. It comprises repeated DNA sequences of 29 nucleotides separated by variable, 32-nucleotide spacer regions derived from segments of ‘captured’ viral DNA. Immunity to infection is acquired when DNA from an invading virus is recognised by a Cas protein complex, which integrates a new repeat-spacer unit corresponding to the virus DNA. The new CRISPR repeat-spacer array is then transcribed into a preCRISPR RNA (pre-crRNA), which is processed into CRISPR RNA (crRNA) and guides the nuclease to destroy the DNA of the invading virus.

Several Cas nucleases have been characterised but the one most widely used in genome editing in plants is the Cas9 nuclease from *Streptococus pyrogenes*. In the native *Streptococcus pyrogenes* system, a trans-activating crRNA (tracrRNA) molecule is also required for Cas9 activation: tracrRNA is complementary to pre-crRNA and forms a crRNA/tracrRNA hybrid, which guides the Cas9 nuclease to its target. As the system has been developed for genome editing, it has been found that the tracrRNA and crRNA can be combined into once molecule called the single guide RNA (sgRNA) or now more commonly just the guide RNA (gRNA). The gRNA can be designed to target any gene sequence, although editing does require the presence of a protospacer-adjacent motif (PAM) comprising three nucleotides with the sequence NGG, in which N can be any of the four bases. The Cas9 nuclease only becomes active when it interacts with a gRNA; when guided to its target, it anchors itself at the adjacent PAM and cleaves the DNA with a double-stranded break. CRISPR/Cas9 is faster, more specific and more adaptable to different situations than MegNs, ZFNs or TALENs. Thus, the CRISPR/Cas9 system has been rapidly adopted for a wide range of purposes, including medical applications (Zhang 2020) as well as crop improvement.

A second Cas nuclease that is starting to become more popular in plant genome editing is Cas12a (formerly known as Cpf1), which originates from *Acidaminococcus* species (Tang et al. 2017). Even in the native system, Cas12a only requires a single RNA molecule to work, and it leaves sticky rather than blunt ends, which may make it more amenable to knock-in applications requiring DNA to be inserted into the cleavage site. It also has a different PAM site to Cas9, comprising YTN, where Y is a pyrimidine (thymine or cytosine) and N is any base.

CRISPR/Cas9 was first shown to work in plants in 2013 (Shan et al. 2013) and, not surprisingly, disease resistance was one of the first traits to be targeted using the new technology. The citrus (C*itrus sinensis*) gene, *CsLOB1*, for example, has been edited for citrus canker (*Xanthomonas citri*) resistance (Peng et al. 2017), while the cucumber (*Cucumis sativus*) gene *eIF4E* has been edited to impart broad virus resistance (Chandrasekaran et al. 2016). Tobacco has been edited for resistance to *Yellow dwarf virus* (Baltes et al. 2015) and tomato (*Solanum lycopersicum*) has been edited for resistance to *Yellow leaf curl virus* (Ji et al. 2015) and powdery mildew (Nekrasov et al. 2017).

Climate resilience/abiotic stress tolerance has also been a target. The maize gene, *ARGOS8*, for example, has been edited to impart drought tolerance (Shi et al. 2017), while knocking out a NAC transcription factor, *OsNAC041*, in rice using CRISPR/Cas9 increased salt sensitivity, showing *OsNAC041* to be involved in salt stress responses (Bo et al. 2019). Similarly in tomato, knocking out the mitogen activated kinase gene, SlMAPK3 in tomato led to reduced drought tolerance (Wang et al. 2017).

CRISPR/Cas9 has also been used to improve quality, increase nutritional content and impart health benefits. Editing of the *NOR* gene in tomatoes, for example, has been shown to delay fruit ripening and softening (Gao et al. 2020), while lycopene content has been increased over five-fold by knocking out *SGR1, LCY-E, BLC, LCY-B1* and *LCY-B2* genes (Li et al. 2018).

Several genes in rice have been edited to improve yield, including, for example, *DEP1,* which affects panicle size (Zhang et al. 2023). In barley, CRISPR/Cas9 has been used to knock out the *SDP1* lipase gene (Eastmond 2006; Kelly et al. 2013), resulting in the accumulation of storage oil in the leaves and stems and raising the metabolisable energy content of the fodder from the edited plants by > 0.5 MJ/kg dry mass. Meanwhile, colleagues at the John Innes centre in the UK have used CRISPR/Cas9 to knock out the *VRT-A2* gene of wheat, resulting in increased grain size (described as ‘bigger, bolder grains) (Adamski et al. 2021). The edited line has already undergone field trials with a view to translating the bigger grain phenotype into increased yields, although data from the field trials have not been published yet (Cristobal Uuay, John Innes Centre, UK, personal communication).

In our own lab, we have used CRISPR/Cas9 to knock out the asparagine synthetase-2 (*TaASN2*) gene of wheat (Raffan et al. 2021, 2023; Kaur et al. 2024). The reason for doing this was that wheat grain accumulates high concentrations of free (soluble, non-protein) asparagine in the grain (Curtis et al. 2009), and this is converted to the toxic and probably carcinogenic contaminant, acrylamide, during high-temperature cooking and processing, including everyday baking and toasting (reviewed by Kaur and Halford 2023). Wheat has five asparagine synthetase genes, *TaASN1*, *TaASN2*, *TaASN3.1*, *TaASN3.2* and *TaASN4* (Xu et al. 2018). Of these, *TaASN2* is by far the most highly expressed in the grain while not being expressed anywhere else in the plant (Gao et al. 2016), making it an obvious target. Four gRNAs were used simultaneously in a single, polycistronic gene, separated by tRNA sequences (Xie et al. 2015), to increase the chances of success (Raffan et al. 2021). The *Cas9* gene that was used had been codon-optimised for wheat and was introduced into wheat cv Cadenza embryos along with the gRNA gene and a *PAT* marker gene by particle bombardment (Raffan et al. 2021).

Lines derived from two of the edited plants, Line 178 (an A genome *TaASN2* null) and Line 23 (a total *TaASN2* null) underwent a field trial (Europe’s first field trial of genome edited (GE) wheat) in 2021–2022 (Raffan et al. 2023; Kaur et al. 2024). Line 23 showed a significant (*p* < 0.001) reduction of approximately 50% in free asparagine concentration in the grain, compared with its standard Cadenza control, while Line 178 showed a reduction of 14% (Raffan et al. 2023).

This and other editing studies made in wheat involving CRISPR/Cas9 are summarised in Table 1. However, although some of the lines produced in the studies have undergone or are undergoing field trials, as we have described, the only commercial variety to emerge to date is a wheat variety with broad and durable resistance to powdery mildew, which was approved for cultivation and consumption by the Chinese authorities in May 2024. In this variety, the mildew susceptibility locus O (*MLO*) gene is knocked out in all three genomes (Li et al. 2022). There have been a number of studies that have focused on the *MLO* gene; the breakthrough with the Chinese variety is that it also has a large deletion in the B genome, 304 kb upstream of the *MLO-B* gene (Li et al. 2022). This results in the ectopic activation of an adjacent gene, *TMT3*. *TMT3* encodes a tonoplast mocosaccharide transporter, responsible for transporting glucose from the cytosol to the vacuole. It is normally only expressed in spikes due to epigenetic repression, but the deletion in the edited line changes the chromatin landscape around the gene, resulting in release of the epigenetic repression and ectopic expression of the gene. The ectopic expression of *TMT3* reverses the yield penalty normally associated with knocking out the *MLO* genes. The Chinese regulatory authorities appear to have fast-tracked the approval of this new variety, representing a real landmark in the development of crop improvement by CRISPR/Cas.

**Table 1:** Summary of CRISPR/Cas9 based traits introduced into wheat (*Triticum aestivum*)

Gene	Gene function	Purpose	References
*ZIP4*	Chromosome pairing and suppression of crossover between related chromosomes during meiosis	Abiotic stress tolerance: heat stress	Martin et al. (2021)
*DMC1*	Preservation of meiosis during high and low temperatures	Abiotic stress tolerance: heat stress	Draeger et al. (2023)
*MLO*	Resistance to powdery mildew	Disease resistance	Wang et al. (2014) and Li et al. (2022)
*EDR1*	Protein kinase involved in powdery mildew infection	Disease resistance	Zhang et al. (2017)
*PDS*	Phytoene desaturase	Gene editing in wheat: proof of concept	Howells et al. (2018)
*CENH3*	Histone H3 protein	Generation of paternal haploids	Lv et al. (2020)
*NP1*	Putative glucose-methanol-choline oxidoreductase	Male sterility	Li et al. (2020)
*SBEIIa*	Starch branching enzyme	Nutrition: Increased amylose content for resistant starch	Li et al. (2021)
*α-gliadin*	Storage protein	Nutrition: Low gluten content	Sánchez-Leon et al. (2018)
*γ-gliadin*	Storage protein	Nutrition: Low gluten content	Jouanin et al. (2019)
*ASN2*	Asparagine synthetase	Reduced acrylamide formation during cooking and processing (food safety)	Raffan et al. (2021, 2023)
*TabHLH489*	Helix–loop–helix transcription factor	Grain length and weight	Lyu et al. (2024)
*VRT-A2*	Controls grain size	Larger grains	Adamski et al. (2021)
*GW2*	Grain weight/protein content	Yield	Zhang et al. (2018)
*GASR7*	Grain weight	Yield	Zhang et al. (2016)

### Simple knockouts, homology-directed repair and prime editing

As we have described, all of the SDNs used in genome editing introduce mutations by causing double-stranded breaks in the DNA and relying on the cell’s natural, error-prone repair mechanisms to fix them. Double-stranded breaks can be repaired by a number of mechanisms, but a common one is non-homologous end joining (NHEJ), in which the DNA strands on either side of the break are directly re-joined using single-stranded overhangs that often remain on the ends of double-strand breaks. Errors occur when the overhangs on either side of the break do not match up, often resulting in the loss of nucleotides and effectively a deletion in the repaired molecule. Single base pair insertions also occur as a result of the propensity of Cas9 to leave a single nucleotide 5’ overhang at the cleavage site, which is filled in before the DNA strands are re-ligated (Lemos et al. 2018). These mutations may result in the loss of a critical domain in the encoded protein, or a frameshift that alters the amino acid sequence of the protein encoded downstream of the edit, rendering the protein dysfunctional. In this case, the edited gene may be described as silenced or knocked out.

The location of mutations produced by NHEJ are predictable, but the types of mutations (i.e. deletions, insertions or base changes, as well as the size of a deletion) are not. Hence, it is not suited for making specific changes to the DNA sequence, for example to change the nature of the encoded protein in a particular way (Molla et al. 2022). However, that can be achieved by using the system of homology-directed repair (HDR), in which a double-stranded break is repaired using donor DNA. This results in specific DNA bases being introduced into a target DNA sequence. Therefore, HDR can be used for knock in experiments, precise gene replacements and installing complex modifications involving DNA insertions or duplications. For HDR to be successful, the donor templates need to be physically close to the repair site to be available for incorporation into the DNA. This means that HDR has an extremely low efficiency rate and is a difficult, time-consuming and laborious method for use in crop improvement (Molla et al. 2022). Fortunately, therefore, the technique is already being superseded by base and prime editing.

Base editing uses components of the CRISPR system together with enzymes that introduce point mutations into the genomic DNA without making double-stranded breaks. Base editors comprise fusions between a catalytically-impaired Cas9 nuclease and a deaminase. As the gRNA binds its target, a strand of single-stranded DNA loops out, DNA bases within this single-stranded loop are deaminated, and a nick is made in the non-edited strand. The cell’s own mechanisms then repair the DNA using the edited strand as a template. There are three classes of DNA base editor: cytosine base editors that convert a C–G base pair to a T–A base pair, adenine base editors that convert an A–T base pair to a G–C base pair, and CG base editors that covert C–G base pairs to G–C.

Prime editing is even more adaptable than base editing and can produce insertions, deletions and transversions as well as base substitutions. It is similar to base editing but uses a reverse transcriptase instead of a deaminase. During prime editing, it is the forward (non-complementary) strand of DNA that is nicked, while the prime editing guide RNA (pegRNA) is extended to comprise a template for repair incorporating the desired edit, as well as the targeting sequence (Lin et al. 2020; Chen and Liu 2023). Prime editing potentially has diverse applications in crop improvement due to the range of edits that can be introduced.

The differences between standard editing with CRISPR/Cas9, base editing and prime editing are summarised in Table 2. Base editing has been used to introduce point mutations in several plant species, including rice, maize, potato and wheat (reviewed by Chen et al. 2019). However, only substitution mutations can be made with base editing and bystander mutation (the introduction of unintended mutations adjacent to the target site) can occur when multiple A and C bases are in the deamination window (Ni et al. 2023). Prime editing has been used to edit monocots including rice and wheat (Qin and Wei 2020; Lin et al. 2020). However, there have been issues with low editing efficiency, which has been reported to be at less than 10% in wheat (Chen and Liu 2023). Efforts are being made to optimise and tailor prime editing for use with plants, especially those species with larger and more complex genomes, like wheat, and to enable multiplexing and gene stacking. Researchers at the China Agricultural University, for example, have achieved simultaneous editing of ten genes in wheat protoplasts and eight genes in transgenic wheat by mutating the reverse transcriptase and engineering the prime editing protein architecture (Ni et al. 2023). They used a Csy-type ribonuclease 4 (Csy4)-processing system and from this developed an efficient Csy4-mediated multiplex prime editing platform (CMPE). This system is more precise, allows for more diverse mutation types simultaneously and has wider adaptability. Meanwhile, researchers at the Chinese Academy of Sciences have developed PlantPegDesigner, an automated pegRNA design platform based on experimental data of prime editing in rice (Jin et al. 2023). They optimised the pegRNA by designing the sequence according to melting temperature, using dual-pegRNAs and engineering prime editors for enhanced editing efficiency using a reporter system and amplicon sequencing. This system was used to produce prime edited rice and wheat using both single and dual pegRNAs with higher editing efficiencies.

**Table 2:** A summary of the differences between standard CRISPR/Cas9 editing, base editing and prime editing

Method	Enzyme	Single/double stranded break	Donor DNA required	Reaction	Edits produced
Standard CRISPR	Cas9 nuclease	Double	Cleavage—no, Knock-in—yes (via NHEJ/HDR)	NHEJ/HDR	NHEJ: insertions/deletions (various lengths) HDR: precise insertion
Base editing	Cas9 nickase	Single (complementary strand)	No	Deamination	Controlled base substitution, CG → TA, CG → GC, AT → GC
Prime editing	Cas9 nickase	Single (non-complementary strand)	No	Reverse transcription	Any substitution, insertion or deletion

*NHEJ* non-homologous end joining, *HDR* homology directed repair

These methods are still being developed, but currently prime editing is more versatile and efficient than CRISPR HDR/NHEJ and base editing, and the range of mutations that can be produced make it widely applicable to many different situations. On the other hand, base editing is more efficient than prime editing at producing point mutations (Lin et al. 2020). Therefore, the technique to be implemented should be carefully considered based on the organism, the system being used and the desired edits.

## Delivery methods and the generation of non-GM, genome edited (GE) plants

Editing via a SDN requires the nuclease to be inside a plant cell and the easiest way to achieve this is to transform the plant with a gene encoding the nuclease. In CRISPR/Cas, this has to be accompanied by a gene encoding the gRNA that will activate the Cas nuclease and direct it to its target. A gene encoding a marker gene, such as bialaphos resistance (*BAR*) or phosphinothrycin acetyl transferase (*PAT*), which impart tolerance to herbicides based on phosphinothrycin, is also usually included. In other words, there is usually a GM step to genome editing using SDNs, with *Agrobacterium*-mediated transformation or particle bombardment the most widely-used transformation methods, and multiple transgenes may be involved.

We discuss the regulatory situations in different parts of the world in the next section. However, all regulatory authorities that distinguish between GM and GE plants require all transgenes to have been removed before a plant is granted GE status for regulatory purposes. This is important because these authorities all require GE varieties to undergo a less onerous risk assessment process than that required of GM varieties. Once editing has been achieved, of course, the transgenes encoding the editing machinery are no longer required and can be segregated away, usually by selfing edited individuals and screening progeny for plants that are edited but do not carry the transgenes. However, the number of successfully edited plants may be small due to the relatively low efficiency of the editing process (Laforest and Nadakuduti 2022), while the proportion of progeny that are transgene-free may also be low if multiple transgenes are involved. It may be possible to include all of the transgenes in a single plasmid for transformation, but this may make the plasmid too large for particle bombardment. *Agrobacterium*-mediated transformation may be preferable to particle bombardment in such a situation. It also tends to result in a lower number of transgene copies being inserted into the host genome, and is regarded as having less risk of plasmid fragmentation and the insertion of incomplete transgenes or fragments of plasmid backbone that may be difficult to screen for. However, while the use of *Agrobacterium*-mediated transformation in cereals has come a long way in recent years, particle bombardment remains the routine choice for species such as wheat.

Non-GM methods of genome editing with SDNs are being developed. One of these involves transient rather than stable expression of the genes required for the editing process (in other words the genes are introduced into the cell and are briefly expressed but do not insert into the host genome), and this has already been demonstrated to work in wheat (Zhang et al. 2016). Another involves bombarding cells with a Cas nuclease protein and gRNA, so no DNA is involved at all (Toda et al. 2019).

## Regulations covering the commercialisation and marketing of GE crops and crop products

### The global situation

In April 2016, the United States Department of Agriculture (USDA) announced that it would not regulate a mushroom that had been edited using CRISPR/Cas9 to knock out a polyphenol oxidase gene (*Ppo*). Essentially what the USDA was saying was that it did not consider the edited mushroom to be a genetically modified organism (GMO) and, therefore, the regulations governing the use of GMOs in agriculture did not apply. Polyphenol oxidase is responsible for the browning/bruising that occurs when fruits, tubers, mushrooms and other fresh foods are cut open or suffer an impact, such as when dropped or handled roughly during transport. As with the high oleic acid soybeans that we described previously, crop varieties with a similar trait but engineered using RNAi were already on the market, including potatoes and apples. However, these were and are regulated as GMOs.

To our knowledge, the application regarding the mushrooms was made as a test case and the variety has not been marketed. However, the decision opened the way for GE crops to be developed and marketed in the USA and, importantly, gave GE crops a competitive advantage over GM crops. The only stipulation applied by the USDA was that the new variety must not contain a transgene; however, the USDA considers plants that have been genetically modified with the editing machinery but in which the transgenes involved have been segregated away to be non-GM.

Many countries in the Americas and Asia followed suit. In some cases, these were countries that were already growing GM crops, and the experience of regulators and the food supply chains in those countries in using GM crops and their products, from plant breeders and farmers through to processors, retailers and, to some extent, consumers, undoubtedly increased confidence in the new technology. However, not all of them were: Japan, for example, which has been implacably opposed to the cultivation and use of GM crops from the start, approved the marketing of GE tomatoes in 2021. These were produced by Sanatech seeds, a spin-out company from the University of Tsukuba, and had been edited with CRISPR/Cas9 to contain high concentrations of ɣ-aminobutyric acid (GABA) (Ezura 2022). The benefits claimed for these tomatoes for consumers include reduced blood pressure, improved sleep and better skin quality. They are a great example of how new genome editing technologies can be implemented to address nutrition issues, and perhaps the fact that they were designed for consumer benefit has aided their acceptance. Nevertheless, the contrast between the approach taken by the Japanese authorities to these tomatoes and their approach to GM crops is remarkable. The Chinese authorities quickly followed suit, approving the use of genome editing for crops in 2022, indicating that concerns over future food security contributed to the decision. This led to the fast-track approval of the mildew-resistant wheat variety that we have already described and which is now on the market (Gao 2021; Li et al. 2022). However, some countries or jurisdictions that have not allowed the cultivation of GM crops to a great extent to date have found the GE crop issue more difficult to deal with.

### The European Union (EU)

The authorisation procedure for cultivation of a GM crop in the EU, or for its import for use in food or animal feed, is based on a safety assessment carried out by the European Food Safety Authority’s Panel on Genetically Modified Organisms, or GMO Panel. The opinion of the GMO panel is then considered by the European Commission, which drafts a decision that is then voted on by the Standing Committee on Plants, Animals, Food and Feed (PAFF). PAFF is a political committee, not a scientific one (the EU is unique in having a political stage in the process) and has rejected almost every application for authorisation to cultivate a GM crop in the EU. As a result, there are only two GM crops currently approved for cultivation: MON810 maize, including some derivative varieties, and the Amflora potato. MON810 was developed by Monsanto and is resistant to the European corn borer and some other insect pests because it expresses an insecticidal Cry protein from a bacterium, *Bacillus thuringiensis* (de Maagd et al. 1999). This so-called Bt technology has been used in many species and varieties around the world and continues to be one of the most successful GM crop technologies developed to date, but MON810 and its derivatives are the only ones allowed to be cultivated in the EU. It was authorised in 1998. The Amflora potato was developed by BASF and contains starch comprising almost entirely amylopectin, with very little amylose, due to suppression of a granule-bound starch synthase (*GBSS*) gene (Visser et al. 1991) by RNAi. Amflora was mired in the EU’s approval process for more than a decade before finally being authorised for cultivation in 2010. Ironically, BASF closed down its European biotech operation soon afterwards and Amflora never made it to market. To our knowledge, it is currently not available anywhere.

Note the 12-year gap in approvals between MON810 and the Amflora potato, and the fact that, to our knowledge, no-one has sought authorisation to cultivate a new GM crop in the EU since. That is not to say that GM crops are not used in the EU; on the contrary, the EU imports millions of tonnes of GM soybean, maize and cotton every year, mostly for use in animal feed, with companies involved in crop biotech focussing on obtaining authorisation for import for food and feed use, which is much easier to obtain, rather than cultivation. It is fair to say, therefore, that the GM authorisation process in the EU is dysfunctional (Halford 2019).

The EUs regulations on GM crops are set out in Food and Feed Regulation (EC) No 1829/2003 and Regulation (EU) No 503/2013. Both regulations draw on Directive (EC) No 2001/18 from 2001, which defines a GMO as an organism in which the genetic material has been altered in a way that does not occur naturally. This extraordinarily broad definition of a GMO meant that crop varieties produced by chemical or radiation mutagenesis, which had been around for decades before the GM regulations were drawn up, had to be given an exemption. This came in the form of the Mutagenesis Exemption, set out in Annex IB of Directive 2001/18, which stated that while crop varieties carrying mutations induced by chemical and radiation mutagenesis were GMOs, as defined by the EU, they did not have to go through the GM risk assessment and approval process before cultivation and marketing.

Genome editing, of course, was developed after the GM definition and regulations had been drawn up. There has been a lot of discussion in the EU and indeed the UK about whether or not GE plants are GMOs. As we state in the previous section, many regulatory authorities around the world consider them not to be. However, they clearly fall within the EU’s absurdly broad definition of a GMO, just like the products of older forms of mutagenesis do. Nevertheless, prior to 2018, the hope and expectation amongst scientists and breeders was that plants produced by the targeted mutagenesis methods of genome editing would get the same exemption from the GM risk assessment and approval process as plants produced by the older, completely random methods of chemical and radiation mutagenesis. Indeed, a number of ways in which GE crops could be introduced into the EU were proposed by scientists (Jorasch 2020; Eriksson et al. 2018, 2020; Bruetschy 2019). These hopes were dashed in 2018 when the European Court of Justice (ECJ) issued a ruling on Case 2018C-528/16.

Case 2018C-528/16 resulted from proceedings brought jointly by Confédération Paysanne, a French farmers’ union, and eight other organisations, against the French government. The plaintiffs sought an annulment of the exemption from GM regulations for organisms obtained by mutagenesis. The ECJ judges confirmed that all plants obtained by any form of mutagenesis breeding are GMOs as defined by Directive 2001/18, but rejected annulment of the Mutagenesis Exemption. However, they stated that the exemption only applied to organisms obtained by methods of mutagenesis that have conventionally been used in a number of applications and have a long safety record; in other words, chemical and radiation mutagenesis but not genome editing. In our view this was a scandalous judgement that completely ignored the scientific consensus on genome editing. It meant that there was no distinction between GM and genome editing as far as risk assessment and authorisation was concerned, and genome editing for crop improvement in the EU fell into the same moribund state as GM.

Eight years on from that decision and the EU is reviewing its stance on genome editing technologies. In July 2023, the European Commission proposed new legislation and categorisation that would take GE crops out of the GM risk assessment and authorisation regulations. It used the term ‘New Genomic Technologies’ (NGT) plant, defined as a genetically modified plant obtained by targeted mutagenesis, *cis*genesis, intragenesis or a combination thereof, on the condition that the NGT plant does not contain any genetic material originating from outside the breeders’ gene pool, including DNA that temporarily may have been inserted during its development. Two categories of NGT plants were proposed, one for edited plants carrying genetic changes that could occur naturally or via conventional breeding techniques, the other for all other GE plants. A new authorisation process would be drawn up for Category 1, while Category 2 would still be treated in the same way as GM plants. Category 1 plants must differ from the parent plant by no more than 20 genetic modifications of the following types: substitution or insertion of no more than 20 nucleotides; deletion of any number of nucleotides; targeted insertion of a contiguous DNA sequence; substitution of an endogenous DNA sequence with a contiguous DNA sequence from the breeders’ gene pool of the same species, where the modification does not interrupt an endogenous gene; targeted inversion of a sequence of any number of nucleotides; any other targeted modification of any size, on the condition that the resulting DNA sequences already occur in the breeders’ gene pool.

There does seem to be a determination within the European Commission to bring this legislation forward, but the process is painfully slow and has considerable opposition within the European Parliament. It was interrupted by the recent European Elections in June 2024, and it is not clear when the proposals are likely to be brought before the new parliament.

### The United Kingdom

The United Kingdom (UK) officially left the EU on 31st January 2020. The EU’s laws on GM and GE crops rolled over into UK law on that date, but the UK government quickly began the process of making changes. First, it passed what is called a Statutory Instrument to amend the Genetically Modified Organisms (Deliberate Release) Regulations 2002(3). This was not new legislation but changed the way that the GM legislation was applied in practice in the UK. It introduced the concept of a ‘Qualifying Higher Plant’ (QHP), a term applied to a GE plant that no longer contains a transgene, and includes a different and much simpler licensing process for field release of a QHP for research purposes. The process requires a short notification to be made to the UK’s Department for the Environment, Food and Rural Affairs (DEFRA) 21 days before the release of the QHP, with responsibility for ensuring that the plant complies as a QHP resting entirely with the applicant. The new system came into effect in March 2022 and was probably the first positive step in the regulation of agricultural biotechnology in the UK for a quarter of a century.

This was followed a year later by entirely new legislation in the form of the Genetic Technology (Precision Breeding) Act, which was given royal ascent and written into English law on 23rd March 2023. This Act introduces the concept of a Precision Breeding Organism (PBO), defining a PBO as an organism that has been modified using technology to introduce targeted genetic changes that could have occurred naturally or via traditional breeding methods. The definition of a PBO, like that of a QHP, includes everything currently possible through genome editing, as long as no transgene is present. Notably, PBOs that have been approved for marketing and foods made from them will not have to be labelled. The term PBO for research will replace QHP for the field release of plants for research purposes only.

The Genetic Technology (Precision Breeding) Act becoming law has been described as marking the start of a new era for English farming (Caccamo 2023). However, it was not the end of the process because secondary legislation (more Statutory Instruments) had to be put in place to set out how the new regulations would work, including how the UK’s Food Standards Agency (FSA) would engage with it. This secondary legislation was approved by the UK Parliament in March 2025.

The process that has been put in place requires an application for a new crop variety to be granted PBO for marketing status to be submitted to DEFRA and considered by the Advisory Committee on Releases to the Environment (ACRE), an expert scientific committee. At this point it is unclear what information ACRE will ask for to make its decision, including what evidence it will require that the new variety contains no transgenes. Once PBO status is approved by ACRE, the FSA will decide whether the PBO can be approved for food and feed use. This decision will be made by another expert committee, the Advisory Committee on Novel Foods and Processes (ACNFP). The FSA conducted a public consultation on the matter in 2022 (FSA 2022) and proposes to categorise PBOs as Tier 1 or Tier 2. Tier 1 PBOs should be approved rapidly, but Tier 2 PBOs, which would include those with ‘significant’ changes in composition compared with their ‘traditionally bred’ counterparts, will undergo a bespoke risk assessment process.

Clearly, there remains uncertainty over how this system will work, and plant breeders in the UK will not develop GE varieties while that uncertainty remains. The situation is also clouded by the fact that decisions on adoption of the Genetic Technology (Precision Breeding) Act are devolved to the Scottish and Welsh Parliaments and the Northern Ireland Assembly, and these bodies have so far all rejected it, so the new regulations only apply in England (UK Parliament, Parliament Bills 2023).

## Concluding remarks

Genome editing is still a very new technology and could be described as the second biotechnological revolution in crop improvement after the GM revolution of the last century. Rapid advances are still being made in its application, particularly in methods involving CRISPR/Cas. That makes it difficult for regulators to keep up, but several countries have already put systems in place that have enabled GE crop varieties to be commercialised. Anecdotally, those countries are seeing a broad range of companies, from established plant breeders to SMEs, spinouts and start-ups getting involved, with innovators, entrepreneurs and investors keen to move into the GE space.

Many of those countries have been growing GM crops already and have learnt from their experience with that technology to develop their approach to GE crops. Farmers, food businesses, retailers and consumers in those countries may be less fazed by the introduction of another new technology in plant breeding. This does not apply to all of the countries who are adopting GE varieties, however, and the enthusiasm of Japan, for example, which has never allowed the cultivation of GM crops, is particularly striking.

Other countries and jurisdictions like the UK and EU that have never allowed cultivation of GM crops are finding the development of regulations for GE crops more difficult. Both the UK and the EU are moving towards systems that should allow the commercialisation of GE crops, but progress has been painfully slow and it remains to be seen how easy it will be to navigate those systems when they are in place. Our advice is that the imposition of a regulatory system that is in any way obstructive will result in plant breeders turning their backs on GE in the same way that they have done on GM. It is worth remembering that the EU (and by default the UK) has a system in place for the risk assessment and authorisation of GM crops, but it is so tortuous that no application has been made for authorisation to cultivate a new GM crop in the EU for a decade and a half.

It could be argued that EU regulations on GM crops simply reflect the antipathy of their consumers to GM, and it is vital that lessons are learnt from the debacle of the GM debate in the 1990s to make sure that GE crops do not incite the same hostility. The EU has made a bad start in that by describing GE crops as a type of GM crop, but it is shackled by the absurdly broad definition of genetic modification in Directive (EC) No 2001/18. We believe that definition was never fit for purpose and should be abandoned. In our experience, consumers consider GM crops to be those containing transgenes, and they understand the distinction that GE crops being brought to market do not.

One thing that was missing in the GM debate in the EU in the 1990s was a discussion of the benefits and potential of the technology. This must not be allowed to happen for GE crops. We are in the middle of a dire environmental crisis that will undoubtedly threaten food security in the coming years and decades. Genome editing has the potential to improve crop yield, nutritional value, quality, food safety, disease resistance, insect resistance and climate tolerance. It represents an opportunity that we cannot waste.

## Abbreviations

ACNFP: UK’s Advisory Committee on Novel Foods and Processes
ACRE: UK’s Advisory Committee on Releases to the Environment
Cas: CRISPR-associated nucleases
CMPE: Csy4-mediated multiplex prime editing platform
CRISPR: Clustered regularly interspaced short palindromic repeats
crRNA: CRISPR RNA
DEFRA: UK’s Department for the Environment, Food and Rural Affairs
ECJ: European Court of Justice
EU: European Union
FSA: UK’s Food Standards Agency
GABA: ɣ-Aminobutyric acid
GE: Genome edited
GM: Genetically modified
GMO: Genetically modified organism
gRNA: Guide RNA
HDR: Homology-directed repair
IPCC: Intergovernmental Panel on Climate Change
MegNs: Meganucleases
NGT: New genomic technologies
NHEJ: Non-homologous end joining
ODM: Oligonucleotide-directed mutagenesis
PAFF: EU Standing Committee on Plants, Animals, Food and Feed
PAM: Protospacer-adjacent motif
PBO: Precision breeding organism
pegRNA: Prime editing guide RNA
pre-crRNA: PreCRISPR RNA
QHP: Qualifying higher plant
RDOs: Double-stranded RNA–DNA oligonucleotides
RNAi: RNA interference
SDNs: Site-directed nucleases
SFHR: Small fragment homologous replacement
sgRNA: Single guide RNA
TALEs: Transcription activator-like effectors
TALENs: Transcription activator-like effector nucleases
UK: United Kingdom
USDA: United States Department for Agriculture
tracrRNA: Trans-activating crRNA
ZFNs: Zinc-finger nucleases
